# Supplementary material for: Meat-Free Mondays in Hospital Cafés in Aotearoa, New Zealand
Source: Nutrients. 2023 Nov 16;15(22):4797. doi: 10.3390/nu15224797 (PMC10675120; doi:10.3390/nu15224797)
Supplement: Supplementary file 1 [file nutrients-15-04797-s001.zip › nutrients-2683638-SI.pdf]

Document Supplement S1

Meat Free Mondays Survey

We are researchers at the University of Auckland conducting a survey as part of study to investigate Meat Free Monday Policies at staff and guest cafés in District Health Boards (DHBs) in Aotearoa. We are interested in finding out what you think about these policies, the food provided, the enablers and barriers for these policies and for staff to reduce meat intake.

Please read the HYPE Meat Free Mondays Participant Information Sheet (PIS) below. After reading the PIS, if you choose to answer the questions in this survey, you are consenting to participate in this research. We will not collect any personal information that could identify you unless you agree to provide your email address to be emailed the study results. The survey will take about 5-15 minutes to complete.

Ngā mihi maioha - Thank you for taking part

1. Consent: Do you consent to participate?

Yes (continue survey)

No (end survey)

2. Screening question: Are you 18 years or over?

Yes (continue survey)

No (end survey)

Information on participants:

3. Do you identify as:

Female

Male

Gender Diverse

Prefer not to answer

4. Please indicate your age group

18-24 years

25-34 years

35-44 years

45-54 years

55-64 years

65 years and over

5. Which ethnic group (or groups) do you belong to? Select all that apply.

New Zealand European / Pākehā

Māori

Samoan

Cook Island Māori

Tongan

Niuean

Chinese

Indian

Other

If Other, please specify: ____________________________________

6. What is your role at your selected DHB/Organisation?

Medical doctor

Nurse or Midwife

Allied Health or other health professional

Academic staff

Administration staff

Support staff (e.g., maintenance, cleaners)

Management staff

Other staff

If Other, please specify: ____________________________________

7. Which of these options best describes your eating habits?

Meat eater (I eat meat regularly) (go to Q8)

Flexitarian (I am primarily vegetarian, but occasionally eat fish, or meat, including chicken - less than one portion of red meat/week) (go to Q9)

Pescatarian (I eat fish, but no other meat types) (go to Q13)

Vegetarian (I do not eat meat or fish) (go to Q13)

Vegan (I do not eat any animal products) (go to Q13)

8. How many dinners a week would you typically eat meat? e.g., red meat, chicken etc.

Less than 1 time per week

1–2 times per week

3–4 times per week

5–6 times per week

7 or more times per week.

9. Are you actively cutting back on meat, e.g., red meat, chicken etc.?

Yes (go to Q10)

No (go to Q12)

10. What is the reason(s) you are cutting back on meat? (Select all that apply)

Health

Saving money

Environmental concerns

Animal welfare

I enjoy plant-based dishes

Other (please specify) _____________

11. If you are incorporating more Meat Free meals, what challenges are you having?

(Select all that apply)

Lack of skills and knowledge to cook tasty Meat Free meals

Making the meals culturally appropriate

Concerns about health

Lack of familiarity

Attitudes of friends, whānau, and/or family

Other, (please specify) _____________

Any further comments: ___________(free text)

12. What is the reason(s) that you are not considering cutting back on the amount of meat that you eat? (Select all that apply)

I like the taste of meat

I am familiar with eating meat, it's what I am used to

I think meat is part of a heathy diet

I don't believe reducing meat intake will have any impact on the environment

It's not culturally appropriate to exclude meat

Attitudes of friends, whānau, and/or family

Other (please state)___________________

Any further comments: ___________ (free text)

13. 'Meat Free Mondays' is a global meat reduction campaign that encourages people to reduce their meat consumption for their personal health and the health of our planet. Are you aware of the Meat Free Monday campaign?

Yes

No

14. Some hospitals in Aotearoa are trialing or have implemented a Meat Free Monday policy at staff and guest cafés - where only vegetarian and vegan meals are available on Mondays. Are you aware of the Meat Free Monday policy in NZ

hospitals?

Yes

No

15. How do you feel about the Meat Free Monday policy or a potential Meat Free Monday policy at your hospital?

Very positive

Positive

Neutral

Negative

Very Negative

Any further comments: ___________ (free text)

16. Do you wish to receive a summary of the HYPE Meat Free Mondays study results?

Yes, please provide an email address: ______________________________

No

Ngā mihi maioha - Thank you for taking part
